# A retrospective study on intracranial mixed infection with tuberculous meningitis in Shenzhen, China

**DOI:** 10.1128/spectrum.03747-23

**Published:** 2024-05-20

**Authors:** Mutong Fang, Sinian Li, Zhi Mao, Xuhui Liu, Xiaomin Wang, Shuihua Lu

**Affiliations:** 1 Department of Pulmonary Medicine, Shenzhen Third People’s Hospital, Shenzhen, Guangdong, China; 2 National Clinical Research Center for Infectious Diseases, Shenzhen Third People’s Hospital, Shenzhen, Guangdong, China; Quest Diagnostics Nichols Institute, Chantilly, Virginia, USA

**Keywords:** intracranial mixed infection, tuberculous meningitis, cryptococcal meningitis, HIV

## Abstract

Tuberculous meningitis (TBM) is a prevalent global intracranial infection and the most lethal and disabling form of tuberculosis. TBM with mixed intracranial infections is clinically rare but has a higher mortality rate. To investigate the clinical characteristics of TBM with mixed intracranial infections, demographic and clinical data of TBM and pulmonary tuberculosis (PTB) patients admitted to Shenzhen Third People’s Hospital between January 2015 and October 2022 were collected anonymously. A total of 207 cases of TBM were diagnosed, of which 16 cases (7.73%) were TBM with mixed intracranial infections. The overall mortality rate of TBM cases was 16.4%, while the mortality rate of TBM cases with mixed intracranial infections was as high as 35.7%. Compared to simple TBM cases, TBM cases with mixed intracranial infections had severer clinical symptoms. The percentage of human immune deficiency virus (HIV)-positive TBM cases with mixed intracranial infections reached up to 68.8%. HIV co-infection, CD4^+^/CD8^+^ T-cell counts less than 1, cranial nerve impairment, paralysis, cerebral infarction, PRO less than 450 mg/L, WBC less than 10 × 10^6^ /L, and CL more than 120 mmol/L were risk factors for TBM cases with mixed intracranial infections. Compared to PTB, HIV co-infection, CD4^+^ T cell less than 550 /uL, and age less than 45 years were risk factors for TBM, and TBM was associated with higher mortality rates. Our study provides additional data to better understand single TBM and TBM with mixed intracranial infections. More than two-thirds of TBM cases with mixed intracranial infections were HIV-positive. Clinicians should consider the possibility of multiple infections in people with TBM/HIV co-infection.

**IMPORTANCE:**

TBM can cause severe neurological damage and death, and TBM with mixed intracranial infections can exacerbate the damage and poor prognosis of the disease. TBM with mixed intracranial infections is a rare disease, which has led to an incomplete understanding of its clinical features. This study investigated the clinical features of TBM and its associated factors by comparing the characteristics of TBM with mixed intracranial infections, single TBM and pulmonary tuberculosis. This information will help to improve the understanding of TBM, diagnostic accuracy and treatment outcomes.

## OBSERVATION

Tuberculous meningitis (TBM) is a common intracranial infection worldwide and the most lethal and disabling form of tuberculosis (TB), especially in children and human immune deficiency virus (HIV)-positive patients (1, 2). In 2019, an estimated 164,000 adults worldwide developed TBM, of whom 78,200 died, a mortality rate of 48% (3). Even when treatment for TBM is successful, serious sequelae can occur (4). Although the range of long-term sequelae of TBM is broad, the most common long-term sequelae are physical and developmental disabilities. Neurocognitive deficits can occur in the absence of physical disability and have important psychosocial and educational consequences for children, especially those with immature brains (5).

In addition, the presence of the *Mycobacterium tuberculosis* (*M.tb*) cannot exclude complications from other intracranial infections, such as cryptococcal meningitis, viral meningitis, and syphilis. Mixed intracranial infections were previously thought to be rare, but recent reports have shown an increase in such cases, particularly among acquired immunodeficiency syndrome (AIDS) and other immunocompromised patients (6, 7). TBM with mixed intracranial infections has a higher mortality rate and a greater impact on patient health. This poses a greater challenge to the already difficult-to-treat TBM and raises further concerns about the disease. However, studies and case reports of this disease are limited due to the small number of TBM cases with mixed intracranial infections. This has hindered improvements in the understanding and treatment of this disease. Therefore, this study retrospectively compared the clinical characteristics of 16 cases of TBM with mixed intracranial infection, 191 cases of simple TBM, and 273 cases of pulmonary tuberculosis (PTB), which may be helpful in improving the prognosis of these patients.

The study was conducted at the Shenzhen Third People’s Hospital, China, which is the main TB-designated hospital in Shenzhen. Demographic and clinical data of patients admitted to the TB department of the hospital were collected and recorded anonymously from January 2015 to October 2022. TBM patients underwent cerebrospinal fluid (CSF) examination, imaging, and biochemical and pathogenetic testing. TBM severity was classified by the Glasgow Coma Scale (GCS) score (8) and British Medical Research Council tuberculous meningitis severity (BMRC) grade (9). BD MGIT 960 culture and GeneXpert MTB/RIF (Xpert) (Cepheid Inc., United States) assay (10) were used to detect *M.tb*, while India ink stain (11) and/or the latex agglutination test (12) were used to detect *Cryptococcus*; toluidine red unheated serum test (TRUST) (13) and/or *Treponema pallidum* particle agglutination (TPPA) (SERODIA-TP.PA Fujirebio inc., Tokyo) (14) test were used to detect *Treponema pallidum*; the IgM (LIAISONToxo IgM, DiaSorin S.p.A., Italy) test were used to detect *Toxoplasma gondii* (15); RT-PCR was used to detect cytomegalovirus (CMV) (CMV PCR test kit, DAAN GENE, China) (16), Epstein–Barr virus (EBV) (EBV PCR Test kit, DAAN GENE, China) (16), and HIV (COBASHIV-1, Roche, United States) (17); and bacterial culture was used to detect other pathogenic bacteria. The criteria used to define cases of TBM are primarily based on *M.tb* detected in the CSF in combination with clinical features, imaging findings, and other laboratory tests. TBM with mixed intracranial infections were defined when other pathogens were detected in the CSF, in addition to *M.tb*. To explore the risk factors associated with TBM, an additional 273 PTB cases from the same study period were included, all of which were BD MGIT 960 culture and/or Xpert-positive and without extrapulmonary TB.

Statistical analyses were performed using Stata version 16.0 (StataCorp, College Station, Texas, USA). Categorical variables were described as counts and percentages and analyzed by the chi-squared test and Fisher’s exact test. Univariate and multivariate logistic regression models were used to calculate odds ratio (OR), adjusted odds ratio (aOR) and 95% confidence interval (CI) for factors associated with TBM. Due to the small number of TBM cases with mixed intracranial infections and the incomplete clinical data on these cases, the factors associated with TBM with mixed intracranial infections were only analyzed using univariate logistic regression models. A *P*-value of less than 0.05 indicated statistical significance.

From January 2015 to October 2022, 207 cases of TBM were diagnosed, of which 16 cases (7.73%) were TBM with mixed intracranial infections (Table 1). Among the TBM cases with mixed intracranial infections, 81.3% (13/16) were male, with mean age 34.8 years; 12.5% (2/16) of cases were retreated TB cases; 18.8% (3/16) were multidrug-resistant and rifampicin-resistant TB (MDR/RR-TB); 68.8% (11/16) of patients were co-infected with HIV; 25% (4/16) of patients had symptoms of paralysis; 18.8% (3/16) of patients had cranial nerve injury; and 35.7% (5/14) of patients died. In addition to *M.tb*, viruses were detected in the CSF of 10 TBM cases with mixed intracranial infections (six cases of EBV, two cases of CMV, two cases of both EBV and CMV), spirochetes were detected in three cases (*Treponema pallidum*), fungi in two patients (Cryptococcus), bacteria in one case (*Pseudomonas aeruginosa*), and protozoa in one case (*Toxoplasma gondii*). Three pathogens (*M.tb* and two other pathogens) were detected in the CSF of three patients, all of whom were HIV-positive and one of whom died.

**TABLE 1:** Detailed information on TBM cases with mixed intracranial infections^*a*^

Case ID	Gender	Age	PTB form	MDR/RR-TB	Mixed infection pathogen	HIV co-infection	Malnutrition^*b*^	CD4 +T cell/uL	CD8 +T cell/uL	CD4/CD8	Outcome
1	Male	28	Miliary	+	EBV/CMV	+	+	539	761	0.71	Survival
2	Male	37	Miliary	-	EBV	+	-	46	170	0.27	Survival
3	Male	51	Miliary	-	EBV	+	+	11	179	0.06	N.A.
4	Male	33	Miliary	-	CMV	-	-	58	741	0.08	Death
5	Male	39	N.A.	-	*Cryptococcus*	+	-	153	853	0.18	Survival
6	Female	29	Secondary	-	CMV	+	+	139	973	0.14	Survival
7	Male	55	Miliary	-	EBV/*T. pallidum*	+	-	68	554	0.12	Survival
8	Male	34	Secondary	-	EBV	+	-	145	1886	0.08	Survival
9	Female	32	Secondary	+	*T. pallidum*	+	-	84	197	0.43	Death
10	Male	32	Miliary	-	EBV/CMV	+	-	16	371	0.04	Death
11	Male	37	Secondary	-	*T. gondii*	+	-	224	1017	0.22	Death
12	Male	30	Miliary	-	*Cryptococcus*	+	-	14	145	0.1	Survival
13	Male	29	Secondary	+	*T. pallidum*	-	+	718	315	2.28	Survival
14	Male	30	Miliary	-	*P. aeruginosa*	-	+	76	101	0.75	Death
15	Male	25	Secondary	-	EBV	-	-	N.A.	N.A.	N.A.	N.A.
16	Female	36	Miliary	-	EBV	-	N.A.	N.A.	N.A.	N.A.	Survival

^*a*^ TBM, tuberculous meningitis; PTB, pulmonary tuberculosis; MDR/RR-TB, multidrug-resistant and rifampicin-resistant TB; HIV, human immune deficiency virus; EBV, Epstein–Barr virus; CMV, cytomegalovirus; *T. pallidum*, *Treponema pallidum; T. gondii*, *Toxoplasma gondii; P. aeruginosa*, *Pseudomonas aeruginosa*; N.A., not available.

^*b*^ Person with a BMI of less than 18.5 kg/m^2^ is defined as undernourished (18).

To compare the differences between TBM cases with mixed intracranial infections and simple TBM cases, their demographic and clinical characteristics were analyzed (Table S1). The results showed that the overall mortality rate of TBM cases was 16.4% (29/177), and the mortality rates of TBM cases with mixed intracranial infections and simple TBM cases were 35.7% (5/14) and 14.7% (24/163), respectively. HIV co-infection (OR 30.12, *P* < 0.001), the ratio of CD4^+^ to CD8^+^ T-cell counts less than 1 (OR 18.97, *P* = 0.005), cranial nerve impairment (OR 7.12, *P* = 0.010), CD8^+^ T-cell greater than 320 /uL (OR 6.36, *P* = 0.001), paralysis (OR 6.03, *P* = 0.007), and cerebral infarction (OR 4.28, *P* = 0.028) were risk factors for TBM cases with mixed intracranial infections (Table 2; Table S2). Among the CSF parameters, protein less than 450 mg/L (OR 14.31, *P* = 0.002), white blood cell less than 10 × 10^6^ /L (OR 13.36, *P* = 0.012), and chloride greater than 120 mmol/L (OR 3.45, *P* = 0.034) were also risk factors for TBM cases with mixed intracranial infections.

**TABLE 2:** Significant characteristics associated with TBM with mixed intracranial infections identified by univariate logistic regression analysis^*a*^

Characteristics		Mixed infection TBM (*n* = 16)		Single TBM (*n* = 191)		Total	OR	(95% CI)	*P* value
		N	%	N	%				
Impairment of the cranial nerve	Yes	3	18.8%	6	3.1%	9	7.12	(1.59–31.75)	0.010
Paralysis	Yes	4	25.0%	10	5.2%	14	6.03	(1.65–22.10)	0.007
HIV co-infection	Yes	11	68.8%	13	6.8%	24	30.12	(9.09–99.79)	<0.001
CD8 +T cell <320 (*n* = 164)	No	8	57.1%	26	17.3%	34	6.36	(2.03–19.88)	0.001
CD4+/CD8+ < 1 (*n* = 164)	Yes	13	92.9%	61	40.7%	74	18.97	(2.42–148.8)	0.005
CSF parameters									
WBC <10 × 10^6^ /L (*n* = 205)	Yes	2	12.5%	2	1.1%	4	13.36	(1.75–102.09)	0.012
PRO <450 mg/L (*n* = 205)	Yes	3	18.8%	3	1.6%	6	14.31	(2.62–78.02)	0.002
CL <120 mmol/L (*n* = 205)	No	5	31.3%	22	11.6%	27	3.45	(1.1–10.86)	0.034
MRI									
Cerebral infarction (*n* = 171)	Yes	13	81.3%	78	50.3%	91	4.28	(1.17–15.61)	0.028

^*a*^ TBM, tuberculous meningitis; HIV, human immune deficiency virus; CSF, cerebrospinal fluid; WBC, white blood cell; PRO, protein; CL, chloride; MRI, magnetic resonance imaging; OR, odds ratio; CI, confidence interval; Ref., reference.

To identify factors associated with TBM, clinical data from 273 PTB cases were collected. By univariate logistic regression analysis, HIV co-infection (OR 35.67, *P* < 0.001), CD4^+^ T cell less than 550 /uL (OR 10.94, *P* < 0.001), and age less than 45 (OR 7.16 for age ≤24, OR 3.87 for age 25–44, *P* < 0.001) were risk factors for TBM, and TBM was associated with higher mortality rates (OR 10.52, *P* < 0.001) (Table S3). All risk factors remained associated with TBM by multivariate logistic regression analysis (Table 3), except HIV co-infection, which could not be analyzed because of the small number of cases.

**TABLE 3:** Results of multivariate logistic regression analysis for identification of covariates that were independently associated with TBM^*a*^

Characteristics	aOR	(95% CI)	*P* value
Age ≤44	6.78	(2.61–17.57)	<0.001
Death	8.22	(2.15–31.46)	0.002
CD4 +T cell <550	7.80	(3.37–18.06)	<0.001

^*a*^ TBM, tuberculous meningitis; aOR, adjusted odds ratio; CI, confidence interval.

Tuberculous meningitis accounts for 70%–80% of cases of neurotuberculosis (19) and is the most deadly and disabling form of TB. This study showed that the overall mortality rate of TBM cases was 16.4%, while the mortality rate of TBM cases with mixed intracranial infections was as high as 35.7%. Compared to simple TBM, TBM cases with mixed intracranial infections had severer clinical symptoms and higher rates of cranial nerve impairment, paralysis, and cerebral infarction. The percentage of HIV-positive TBM cases with mixed intracranial infections was as high as 68.8%. Compared with simple TBM, TBM cases with mixed intracranial infections had a significantly higher proportion of WBCs less than 10 × 10^6^ /L and PRO less than 450 mg/L in CSF, but a significantly lower proportion of CL less than 120 mmol/L, which may be clinical features of TBM cases with mixed intracranial infections.

HIV-infected patients are at increased risk of developing TBM, especially in the more severely immunosuppressed stages (20). A previous study reported an estimated prevalence of 30% for HIV in patients with confirmed TBM and 12.1% in patients with suspected TBM (21). HIV co-infected TBM patients also have higher mortality rates than HIV-negative patients (22). A similar phenomenon was found in this study, where the proportion of HIV-positive TBM cases with mixed intracranial infections was significantly higher than that of simple TBM cases. About half of HIV-positive patients develop encephalitis and neurobehavioral deficits, which also cause structural and functional damage to the blood–brain barrier (23–26). This may be an important reason for the high frequency of mixed intracranial infection TBM in HIV-positive patients.

This study found that viruses, bacteria, fungi, protozoa, and spirochetes had the potential to co-infect with *M.tb* in the cranial cavity. The highest proportion of viral co-infections was found in TBM cases with mixed intracranial infections. Apart from HIV, the most common infection is EBV, which is a human herpesvirus that infects 90%–95% of the adult population. People infected with EBV can carry the virus for life (27, 28). Meningitis caused by EBV infection is more likely to occur in immunocompromised hosts (29). The high percentage of EBV co-infection in this study also indicated immunocompromised patients. CMV, another herpesvirus, was detected in 25% of the TBM cases with mixed intracranial infection in this study. CMV usually causes asymptomatic infections in immunocompetent hosts but can cause end-organ disease in immunocompromised individuals at sites such as the gastrointestinal tract, retina, or central nervous system (30). Other pathogens identified in this study, such as *Cryptococcus* (31) and *Toxoplasma gondii* (32), are common pathogens in immunosuppressed patients. Cryptococcal meningitis caused by *Cryptococcus* is the most common form of meningitis in adults with HIV (31). *Treponema pallidum* is the causative agent of the sexually transmitted disease syphilis, a chronic, multistage human infection with variable clinical symptoms (33). However, meningitis caused by *Treponema pallidum* is rare (34).

In this study, we found that TBM cases with mixed intracranial infections had significantly higher proportions of CSF with WBCs less than 10 × 10^6^ /L and PRO less than 450 mg/L, but significantly lower proportions of CSF with CL less than 120 mmol/L. Leukocytes (leukocytes or white blood cells) are important for the body to phagocytose foreign objects and produce antibodies, as well as they repair tissue damage and fight pathogens and diseases (35). Patients with WBCs less than 10 × 10^6^ /L indicate a possible pathogen infection. Patients with CSF PRO values less than 450 mg/L may be associated with malnutrition or decreased CSF production. Abnormal CSF chloride levels may indicate an underlying neurologic disorder, such as meningitis, encephalitis, or hydrocephalus (36). However, all of these CSF features lack specificity, and further studies are needed to investigate whether the combination of these features can be used to differentiate TBM cases with mixed intracranial infections from simple TBM cases. In addition to higher mortality rates and low CD4^+^ counts, which are known characteristics for TBM, cases less than 45 years of age also had a higher risk of TBM. This is probably due to the fact that these age groups are also the main HIV-infected population in China (37).

In conclusion, our study on mixed intracranial infections in patients with TBM provides additional data to help us improve our understanding of this co-infection. The percentage of HIV-positive TBM cases with mixed intracranial infections was as high as 68.8%. Clinicians should consider the possibility of multiple infections in persons with TBM/HIV co-infection. Our study had some limitations. First, as this was a retrospective study, there were inevitably some missing information. Second, the incidence of TBM is low and the number of cases in this study was limited, making it difficult for small differences to reach statistical significance.

## SUPPLEMENTAL MATERIAL

Supplemental material spectrum.03747-23-s0001.xlsx Tables S1 to S3.

ASM does not own the copyrights to Supplemental Material that may be linked to, or accessed through, an article. The authors have granted ASM a non-exclusive, world-wide license to publish the Supplemental Material files. Please contact the corresponding author directly for reuse.
